# Radiographic assessment of pectoral flipper bone maturation in bottlenose dolphins (*Tursiops truncatus*), as a novel technique to accurately estimate chronological age

**DOI:** 10.1371/journal.pone.0222722

**Published:** 2019-09-26

**Authors:** Ashley Barratclough, Roberto Sanz-Requena, Luis Marti-Bonmati, Todd L. Schmitt, Eric Jensen, Daniel García-Párraga

**Affiliations:** 1 National Marine Mammal Foundation, San Diego, California, United States of America; 2 Radiology Department, Hospital Quironsalud Valencia, Blasco Ibáñez, Valencia, Spain; 3 SeaWorld, San Diego, California, United States of America; 4 U.S. Navy Marine Mammal Program, San Diego, California, United States of America; 5 Fundación Oceanogràfic de la Comunidad Valenciana, Gran Vía Marqués del Turia, Valencia, Spain; Animal Health Centre, CANADA

## Abstract

Accurate age estimation in wildlife conservation is an important diagnostic tool in the interpretation of biological data, necropsy examination, reproductive status and population demographics. The most frequently utilized methods to age bottlenose dolphins (*Tursiops truncatus*) include tooth extraction; counting dental growth layer groups and dental radiography. These methods are inaccurate in dolphins > 13 years old, due to overlapping of the growth layer groups in dolphins and worn teeth. Establishing a non-invasive method of accurately aging bottlenose dolphins across the entire age range is important to long term conservation efforts to understand health status, lifespan, reproduction and survivability. A database of 126 radiographs from 94 dolphins of known chronological age was utilized to establish the stages of skeletal ossification over time. A numerical score from -1 to 8 was assigned to 16 anatomic locations on the pectoral radiograph, to create a formula to estimate age. The most informative areas to evaluate morphologically were the metaphyseal regions of the radius and ulna, and the proximal and distal epiphysis of metacarpals II and III. Third order polynomial regression calculated separate age predictor formulas for male and female dolphins, with females reaching sexual maturity earlier than males. Completion of epiphyseal closure of the long bones correlated with average sexual maturity. Managed care dolphin ages could be properly estimated with decreasing precision from within 3 months in animals < 5 years old, to within 5 years in animals > 30 years old. This diagnostic tool could also be applied to diagnose atypical ossification patterns consistent with nutritional, developmental or growth abnormalities, and identifying subclinical health issues. In conclusion, knowledge of the lifespan and the onset of sexual maturity for each species will allow this model to be applied to other cetaceans, facilitating age estimation via pectoral radiography in future research.

## Introduction

Estimating the age of cetaceans can have large implications in interpreting scientific data of individual health assessments, reproductive status and population demographics. Determining the age of stranded individuals with limited pathology on post mortem examination can provide a reliable insight into the cause of death. Establishing chronological age in cetaceans has proved challenging, due to minimal external signs of aging, with several methodologies currently utilized. The most standardized methodology involves counting tooth growth layer groups (GLGs) in medial longitudinal sections of teeth [1–3]. Tooth extraction is however an invasive procedure and whilst useful in necropsy examinations, in live animals, alternative less invasive methodologies are being investigated. In addition GLGs have been shown to be difficult to interpret in older cetaceans [4], giving misleading age estimations [5, 6]. Overlapping of dentine layers in bottlenose dolphins (*Tursiops truncatus*) at ages > 13 years has caused inaccuracy in estimating the ages of adult animals [1]. GLG methodology is challenging with variation in assessment, expertise and in tooth preparation techniques shown to reduce age estimate accuracy [1]. GLGs have also been used effectively in post-mortem analysis to estimate age in ear bones in manatees (*Trichechus manatus latirostris*) [7], tusks in dugongs (*Dugong dugon)* [8], baleen plates and ear wax plugs in mysticetes [9, 10] and claws in bearded seals (*Erignathus barbatus*) [11]. In some cetaceans e.g. beluga whales, where continuous tooth growth is present the neonatal lines and first GLGs are worn away, therefore age cannot be accurately determined in these cetaceans [12]. Due to the expensive technical equipment, high quality control, limitations to handling and transport of actual animal tissues, experienced expertise required to analyze the data and the poor practical application to live animals, alternative methodologies are warranted. Dental radiography has been used to accurately age dolphins < 10 years old when the tooth pulp to tooth size ratio is accurately correlated with age [13]. Tooth wear prevents this method from being utilized to accurately age adult dolphins.

Forensic methods to estimate age in marine mammals have included bomb radiocarbon age validation, radioisotope 210Pb/226Ra disequilibria, histological evaluation of gonadal maturity and aspartic acid racemization in eye lens nucleus [14–18]. Methods such as telomere length and bone density studies have had some success, however are relatively limited in sample size and access to a population of animals of known chronological age [4, 19, 20]. Poor correlation has been observed in archived samples of bone density analysis with known aged animals [21]. They are often limited to post-mortem samples due to the need for de-fleshed samples to accurately assess bone density, reducing the clinical and practical application of this technique [4]. These alternative methods often rely on other additional information such as body length or GLGs to try and establish correlation between two aging methods rather than being independently accurate. As body size can be misleading in determining age, establishing an independent indicator of age estimation would be highly valuable [22]. Imaging techniques such as micro CT of bone has been used in humans and to assess the pulp to dentine ratio and provide an age indication in odontocete fossils [23], however this is not practical in live cetaceans or in the field [24].

Radiographic evaluation of the degree of fusion of pectoral flipper epiphyseal plates or the degree of fusion of the hyoid complex can estimate the age of stranded cetaceans, however this method has not currently been standardized for accurate chronological age estimation, only age group classification [25, 26]. The advantage of utilizing skeletal ossification is that changes occur progressively in an orderly sequence from fetus to elderly adulthood. Radiographs permit direct comparison of individuals without reliance on genetic assessment or total body size and are intimately related to the sexual maturity of the individual and overall maturation [22]. Stranded marine mammals with bone pathology on post mortem such as fractures, spondylosis or osteomyelitis could be aged radiographically to improve understanding of the pathophysiology of necropsy findings [27, 28]. In addition, this methodology could be applied to aborted neonates to establish the age of the fetus and aid interpretation of the cause of pregnancy failure rather than relying on neonate length for age classification [29].

In humans, skeletal ossification has been used to determine age via both hand and foot radiography [30, 31]. A combination of physical exam, dental exam and radiographs are recommended to diagnose abnormal advanced or delayed maturation in children, as well as for the medico-legal age assessment of individuals. As sexual maturity can vary greatly in age of onset, interpreting age estimation in conjunction with skeletal maturity or dental records can improve accuracy [30]. In humans bone age estimation is within 10 per cent of the chronological age [32].

Assessment of skeletal ossification to establish the extent of bone maturity involves a thorough examination incorporating the size, shape and degree of mineralization of the bone and fundamental knowledge of both endochondral ossification and intramembranous ossification progression [32]. Initial perichondral ossification transforms the long bones into the characteristic dumbbell shape. The diaphysis of the bone forms from the primary ossification center, whereas the secondary ossification centers at the ends of the bone form the epiphyses. The epiphyseal plate is the thin layer of cartilage remaining when the secondary ossification center is progressively ossified. The metaphysis lies between the diaphysis and the epiphysis and is where the growth of the bone occurs [32]. Once osteoblasts stop multiplying and the matrix becomes mineralised, the epiphyseal plate is ossified, fusing with the diaphysis and growth ceases to occur. Bone width increases via intramembranous ossification with skeletal tissue developing from fibrous membrane. As ossification occurs in a predictable order, assessment of the degree of maturation of the epiphyses can give an indication of skeletal maturity or bone age [33].

Cetacean limb anatomy is unique in structure due to the adaptation and evolution from a terrestrial to aquatic environment [34, 35]. Paedomorphic patterns of endochondral ossification have been described in the vaquita (*Phocoena sinus)* and harbor porpoise (*Phocoena phocoena*) to identify morphological changes associated with age, however a standardized approach to enable accurate age estimation is lacking [36]. In the majority of cetaceans previously studied, there is an established sequence of ossification progression pattern from proximal to distal including the radius, ulna, carpal, metacarpal and phalangeal bones. This pattern is remarkably constant, symmetrical and the same for both sexes [25, 37–39]. Physiological delayed perichondral ossification of the phalangeal diaphysis demonstrates shape changes in the proximal diaphysis, progressing from ovoid, to deltoid (triangular) and then dumbbell / rectangular [38]. Species variation has been documented. For example, the striped dolphin (*Stenella coeruleoalba*) ossifies the epiphyses of proximal and distal phalanges as well as metacarpals I through IV unlike the harbor porpoise where ossification of the epiphyses in digits one and five is rare [26, 40]. In addition, the rate of skeletal ossification is sexually dimorphic with females reaching skeletal maturity faster than males [26].

As tooth analysis is a skilled, invasive, expensive, legally complex and time-consuming methodology, coupled with the inaccuracy >13 years old, we hypothesize that an alternative simple and standardized non-invasive technique to age cetaceans such as skeletal ossification is highly advantageous [1, 4, 41]. Improved accuracy of aging techniques will transcend into greater understanding of population aging demographics, epidemiology and species survivability. Our aim is to determine the practical application of skeletal ossification maturity assessment as a tool for age estimation in the bottlenose dolphin by utilizing for the first time a globally heterogenous population of precisely known chronological ages to validate the technique.

## Materials and methods

**Fig 1:**
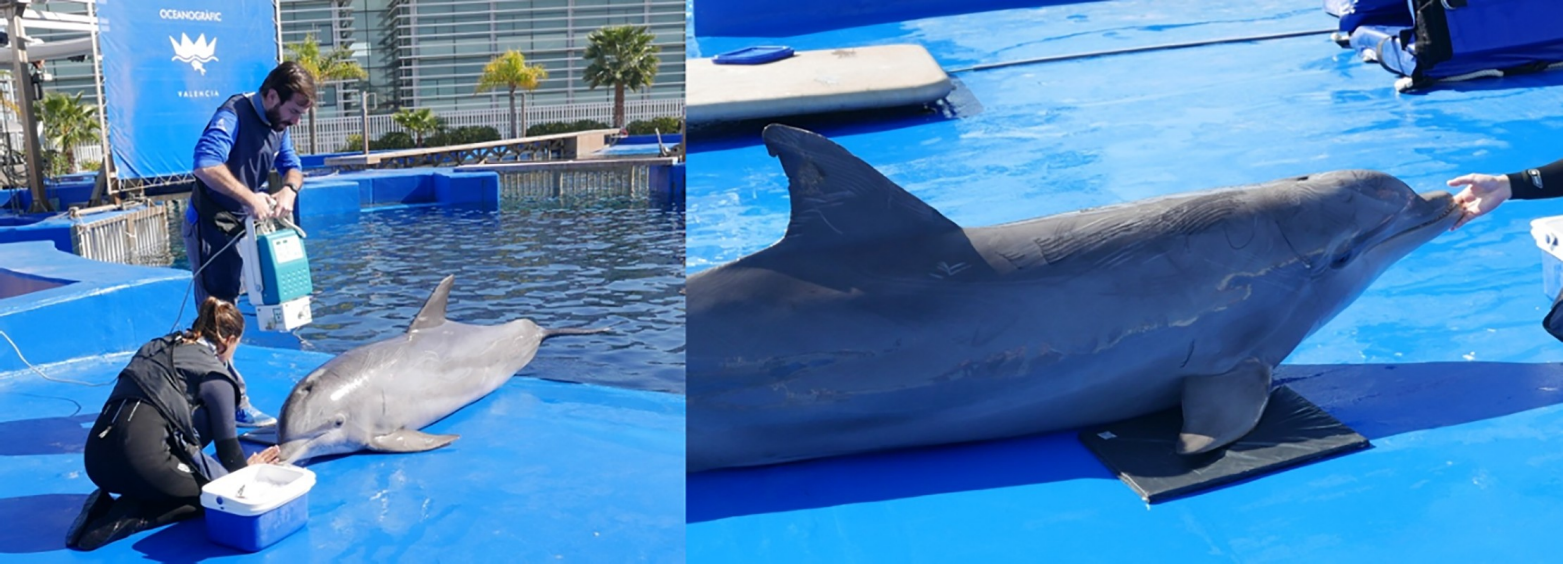
Normal positioning of a bottlenose dolphin to obtain a voluntary radiograph of the right pectoral fin.

To establish the methodology to estimate the age of bottlenose dolphins via radiography three anatomic areas of interest were initially studied in 2005. Radiographs were taken of 12 adult dolphins at Oceanogràfic (Valencia, Spain) of the left lateral-oblique mandible, dorsoventral bilateral pectoral flipper and left lateral caudal vertebral column at the level of the peduncle insertion. Comparison of the osteological changes associated with age in the three different locations demonstrated that the pectoral flipper was the most accurate location for future assessment of maturity indicators [42]. For this study, dorsoventral radiographs were taken from a single pectoral flipper with different X-ray equipment per institution, using optimum settings as close as possible to 70kVp and 6.4mAs. The advent of digital radiography enabled images over or under exposed to be digitally altered to ensure optimum appearance for accurate radiograph scoring and interpretation. Fig 1 shows normal positioning of a dolphin for voluntary pectoral radiography.

Unlike humans, where differences in left and right hand radiographs are observed due to laterality, no significant variation in bone maturation has been observed in preliminary cetacean studies including both pectoral fins, therefore only a single image from each animal and time point was included in the study [20, 25, 43, 44]. Inclusion criteria required the exact age of the animal to be known at the time of the radiograph (or close estimation in animals over 30) and no known health concerns impacting the growth rate of the individual. From 2004 to 2019, 126 radiographs were obtained from 94 individuals from a heterogenous consortium of dolphins which had either been born in human care or resided in managed care for >30 years from 13 different institutions in Europe, Africa, Asia and USA.

Radiographs were reviewed systematically proceeding from proximal to distal, and from cranial to caudal [22]. Each score started at the distal ends of the radius and ulna proceeding to the metacarpals and the associated phalanges systematically. Metacarpal I had poor correlation with age due to a single secondary center of ossification on the proximal epiphysis and was therefore excluded from the scoring system. Five carpal bones are consistently present but again do not show clear differentiated stages of bone maturation and were also excluded from analysis. Additional or fused carpal bones are documented, however are not correlated with age and were excluded from the scoring system. For the bones included in assessment, the centers of ossification progressed through a regular predictable series of changes in form, characterizing the successive stages of maturity and posterior remodeling. Maturity indicators are defined as features of the individual bones that are visible in a dorsoventral radiograph of the flipper that occur regularly and in a definite and irreversible order during aging [22].

**Fig 2:**
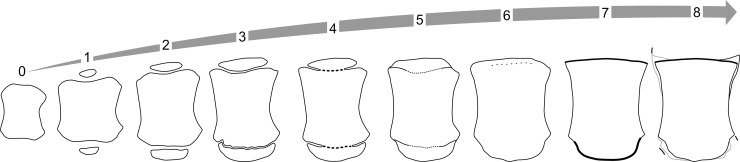
Skeletal ossification scores of the long bones and metacarpal bones. The endochondral ossification progression of the 2nd metacarpal (M2) through 8 different stages. **Stage 0** shows no secondary ossification center with no development at either the proximal or distal ends. **Stage 1** shows the beginning of the secondary ossification centers. Note the difference in width of the ossification center compares to the diameter of the metacarpal and how thin the secondary ossification center is (less than 50% of the surface). **Stage 2** shows an increase in width and depth of the secondary ossification center, that is between 50–80% of the width of the metacarpal. **Stage 3** shows the width of the secondary ossification center to be >80% of the metacarpal physis with a reduction in physeal space but it is still fully open. **Stage 4** shows the formation of osseous bridges between the metacarpal and the secondary ossification centers but consolidation is <50% of the total extension. The ends remain open, fusion is incomplete. **Stage 5** shows the ghost hypermineralized line of where the joining of the secondary ossification center has occurred at least over 50% of the total surface extension. The ends may remain slightly open or fissure be fully consolidated. **Stage 6** full closure of growing plate with complete remodeling of the secondary ossification center with the area showing <50% to no visible ghost line. **Stage 7** shows the flattening of the physeal surfaces of the metacarpal with accentuated pointed edges. **Stage 8** shows degenerative bone or cartilage changes associated with the metacarpal with the presence of either osteolysis or proliferation. In extreme cases calcification of the surrounding cartilage maybe visible.

Scores were assigned to selected long bones (distal radius and ulna, proximal and distal metacarpals II, III, and IV, and phalanges from digits II and III) according to the following criteria, with the principles based on Ogden et al (1981) previous work for scoring bone maturation in dolphin pectoral fins, with two additional categories assigned as shown in Fig 2 [25].

- Score -1 - No primary center of ossification visible.
- Score 0 - Primary center present but no secondary ossification center visible.
- Score 1 - Appearance of epiphyseal ossification center, but size is limited
               to <50% of the width of the latitude of the adjacent metaphysis.
- Score 2 - Appearance of secondary ossification center is well established
- ranging from between 50–100% of the width of the adjacent metaphysis.
               - The physis is clearly evident as a distinct radiolucent space between the secondary center.
               - Secondary ossification center is less mature resulting in irregular depth across the width.
               - Increased radioulnar maturation occurs in a midline to abaxial direction.
- Score 3 - Thinning of the radiolucent physis but no evidence of fusion of
                the metaphysis and the secondary ossification center.
               - Enhancement of mineral density of the subchondral bone at both sides of the physis.
               - Late stage 2 can easily be confused with early stage 3; aim to distinguish between the two stages by critically assessing the uniform nature of the width and if it is irregular in distribution (Stage 2) or even throughout (Stage 3).
- Score 4 - Formation of osseous bridges, in the radius and ulna this starts in
                the midline and proceeds abaxially.
               - Key part of stage 4 is the most abaxial regions are still open.
               - Whether there is the beginning of osseous bridges forming or 90% formed this still qualifies as stage 4.
- Score 5 - Complete closure observed with a faint hyperdense ghost physeal line or
               - epiphyseal scar of at least 50% of the whole plate representing remnants of the juxtaposition of the physeal plates.
               - The epiphyseal plate may not reach full length of the diaphysis but the physis is closed.
- Score 6 - Physeal line is being remodeled with <50% to no evidence of the
                Hypermineralized transverse physeal remnant.
               - The epiphyseal plate covers full length of the diaphysis.
- Score 7 - Complete fusion, no evidence at all of previous ghost physeal plate.
               - Distal ends of the metacarpals and proximal and distal ends of phalanges become flattened with pointed lateral and medial distal corners.
- Score 8 - Arthritic changes apparent including the presence of osteophytes,
                osteolysis, articular calcifications and possible fusion of metacarpal bones or phalanges.

**Fig 3:**
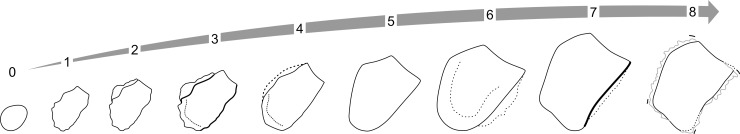
M5 and first phalange of digit 4 delta bone ossification stages. **Stage -1** –Not present. **Stage 0** –Very small oval shape undefined delta shape. **Stage 1** –Axial surface starts to be linear consistent with delta shape. **Stage 2** –one flat surface. Areas or slight irregular perimetral surface typically on the proximal side with a degree of adjacent mineralization which can oppose the epiphyseal surface but not necessarily show definitive osseous bridge formation. Bone density is clearly reduced in peripheral new bone in comparison to the primary ossification center. **Stage 3** –Increased mineralization of adjacent proximal cartilaginous surface, consolidation may not be present. **Stage 4** –Area increases in consolidation and mineralization, slightly less dense than the body of the delta bone. A defined hyper mineralized physeal plate line is present. The abaxial aspect can show the initiation of the new straight lateral border rather than horseshoe shape. **Stage 5** –Density of secondary ossification center is similar to primary bone and initial mineralization present on distal surface with the proximal side showing defined straight lateral border rather than horseshoe shape. Individual variation may result in non-linear borders therefore emphasis should be placed on the secondary ossification centers on the proximal and distal surfaces. **Stage 6** –Smooth proximal surface with matching density to the primary ossification center, distal surface still shows some incongruent mineralization. Physeal line is still visible denoted by dotted line. **Stage 7** –Smooth proximal surface with complete consolidation of the secondary ossification center on both sides and dissolution of the physeal line. **Stage 8** –Degenerative changes present.

The ossification stages from the long bones cannot be directly applied to the delta bones due to the lack of secondary ossification centers. Metacarpal V and the phalanges associated with metacarpal IV are delta bones in bottlenose dolphins [38]. The delta bones ossify at different stages and are therefore inherently valuable in the accurate ageing of older animals. The term delta is used when bones are triangular in shape with a convex longitudinal border and a concave longitudinal border [38]. The following descriptions can be applied to these three bones and are shown in Fig 3:

- Stage -1 –No primary ossification center present
- Stage 0 –Very small ovoid shape, undefined delta shape
- Stage 1 –Part of the surface starts to be linear, consistent with delta shape
- Stage 2 –Non uniform surface with a degree of adjacent mineralization which can oppose the epiphyseal surface but not necessarily show definitive osseous bridge formation. Bone density of the new bone is clearly reduced in comparison to the primary ossification center.
- Stage 3 –Increased mineralization of adjacent axial cartilaginous surface, consolidation may not be present.
- Stage 4 –Area of new bone increases in consolidation and mineralization, slightly less dense than the body of the delta bone. A defined hyperminerlized physeal plate line is present. The abaxial aspect can show the initiation of the other straight lateral border rather than horseshoe shape (delta).
- Stage 5 –Density of secondary ossification center is similar to primary bone and initial mineralization present on abaxial surface with the proximal side showing defined straight lateral opposed border rather than horseshoe shape. Individual variation may result in non-linear borders, therefore emphasis should be placed on the secondary ossification centers on the axial and abaxial surfaces.
- Stage 6 –Smooth axial surface with matching density to the primary ossification center, abaxial surface still shows some incongruent mineralization.
- Stage 7 –Smooth axial surface with complete consolidation of the secondary ossification center on both sides and dissolution of the physeal line.
- Stage 8 –Degenerative changes present

Due to the transition between stages often taking several months to years, half scores were implemented if the radiograph demonstrated a transition into the next stage but it was not complete. For example stage 4.5 would be 95% closure of the plate with a small gap present at the lateral border of the plate, therefore progression has occurred since “stage 4” but it cannot be fully categorized as stage 5. The proximal humerus was not assessed due to inaccessibility to obtain radiographic images within this same plane and difficulty visualizing this in the live animal [25].

The quality of the radiograph is influential on the interpretation of results and accuracy of the age estimation. One of the key features to assess prior to applying the scoring system is the degree of rotation of the carpal cuboidal bones. Any degree of rotation of the pectoral flipper is clearly depicted amongst these five bones and would have a subsequent impact on the interpretation of the growth plates on the metacarpals, where rotation may not be so clearly apparent. Slight rotation can impede the visualization of an epiphyseal line despite the presence of the growth plate and can also alter the appearance of the trabecular pattern across the former epiphysial-diaphyseal junction leading to incorrect scoring [22].

**Fig 4:**
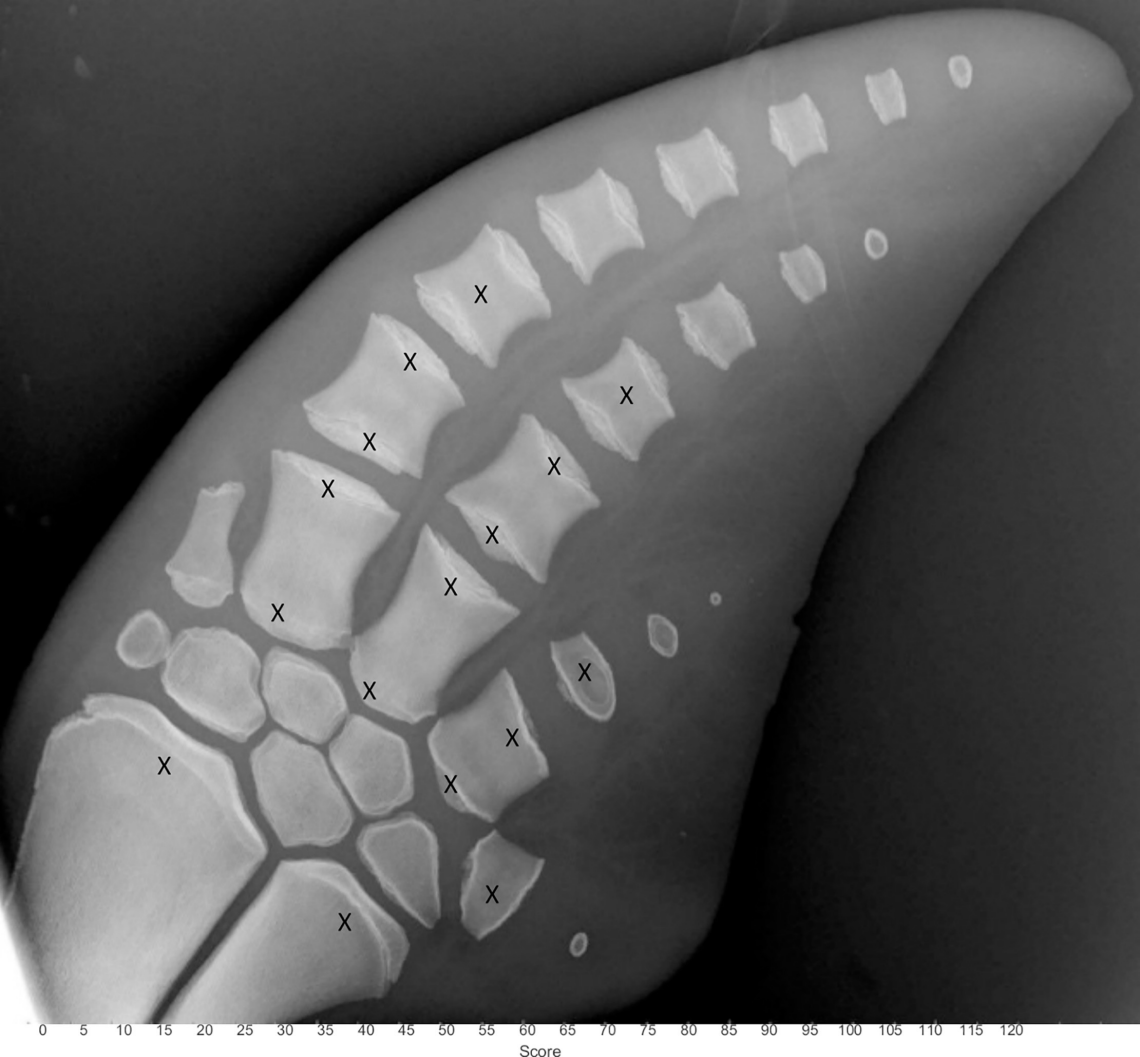
Scoring locations to systematically age a pectoral radiograph in the bottlenose dolphin. Anatomic locations included in the assessment are denoted by X and include the distal surfaces of the radius and ulna, the proximal and distal metacarpals II, II and IV, metacarpal V and the proximal and distal surfaces of the first phalange associated with metacarpals II and III. In addition the second phalange for metacarpals II and III and the first phalange associated with metacarpal IV were given a combined score for the proximal and distal surfaces.

Initially 32 bone surfaces were assessed using a modified scoring system to Ogden et al. and the human recommendation [22, 25]. Pearson’s correlation coefficients were performed to see which of the 32 measurements could be potentially used as positive predictor values in estimating the age. To optimize assessment time and statistical performance, the number locations was reduced via excluding bones showing a correlation with age < 0.80; resulting in 16 final scoring locations (Fig 4).

Each of the radiographs was scored by two independent reviewers in a blind study where the actual age was unknown during the assessment (DGP and AB marine mammal veterinarians). A score of -1 to 8 was given for each of the 16 locations. These 16 individual scores were combined (added up) to give an overall score for each radiograph. Statistical analysis of Wilcoxon Signed rank test was performed to compare inter and intra observer error. A one way repeated measures ANOVA was performed to compare all the scores from all reviewers.

Third order polynomial regressions were performed independently for females and males to assess the relationship between known chronological age of the dolphin and the assigned skeletal ossification score. Datasets were split in training (70%) and test (30%) subsets. In order to obtain the optimal fit for the available data, 1000 iterations were performed, randomly building the training and test subsets for each iteration. Furthermore, to ensure a homogeneous distribution of ages in the training and test samples, four groups were defined: < 5 years, 5–10 years, 10–20 years and > 20 years, so that the randomization always included 70/30% of cases from each group. Model fit was assessed using R^2^, adjusted R^2^ and root mean squared error (RMSE). The results of the test phase were assessed using RMSE.

The digital bone age photographic atlas was created with the best quality image for a given age and sex. The standards were grouped into age categories based on the variability for a given age with increased variation occurring in the earlier growth stages. The intervals between groupings approximate one standard deviation for skeletal maturity at that chronological age. The range of ages is from– 6 months (aborted fetus) to 40 years old in females and– 5 months (aborted fetus) to 58 years old in males.

## Results

A total of 126 radiographs were evaluated in the study; 60 female radiographs from 42 different females, and 66 male radiographs from 52 individuals.

**Fig 5:**
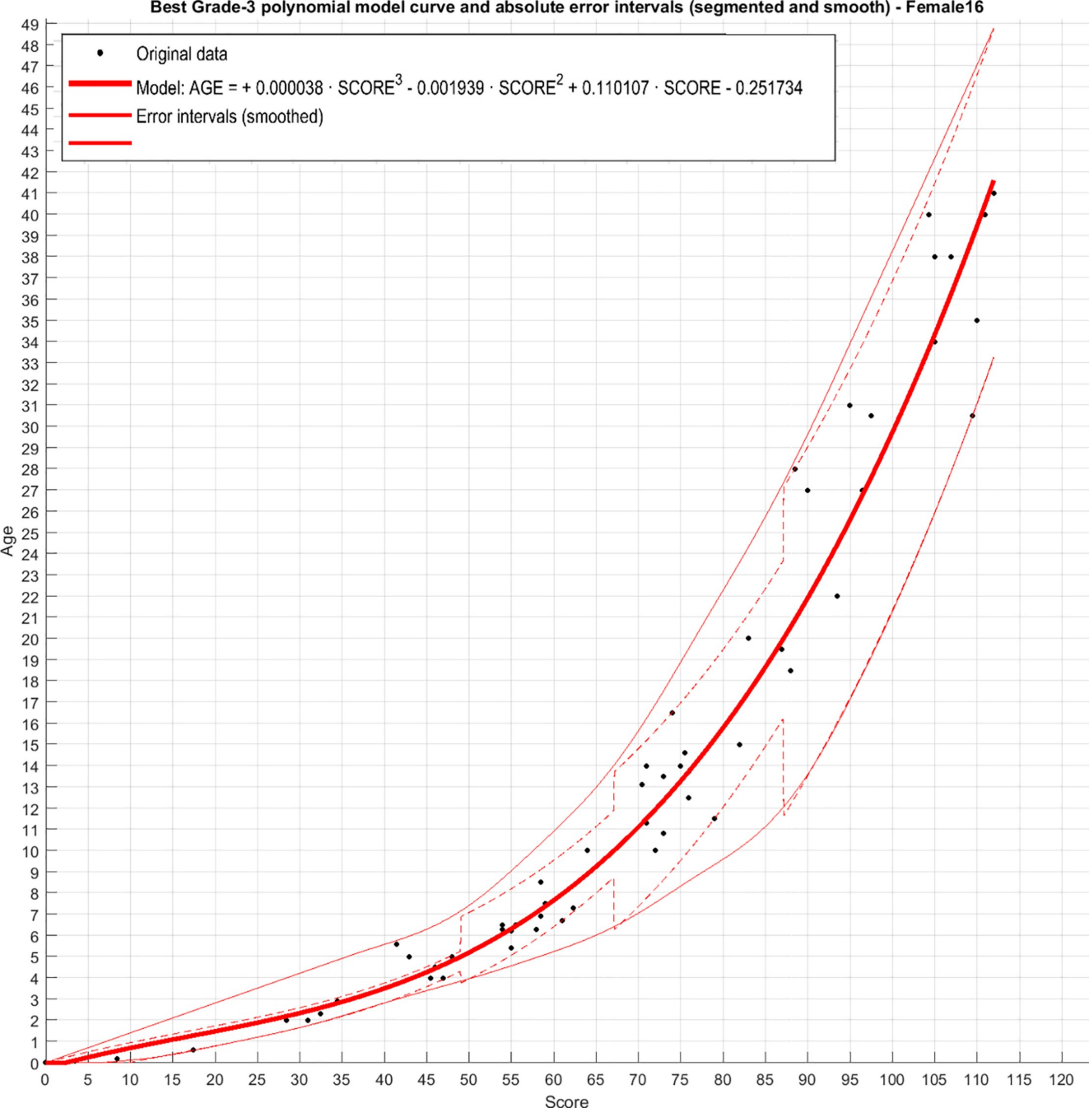
Grade 3 polynomial of score against age (years) in female dolphins with error intervals per age category.

**Fig 6:**
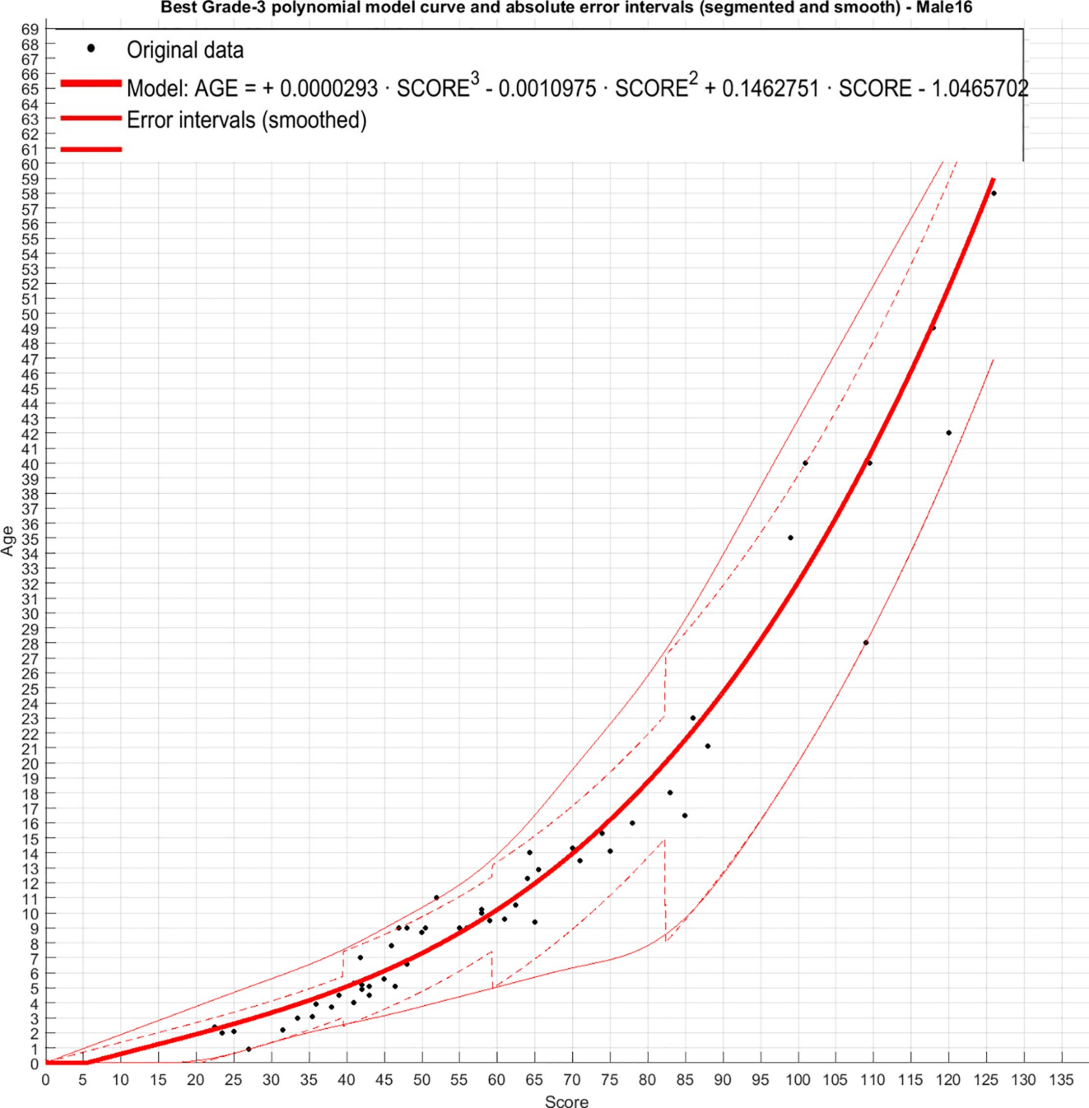
Grade 3 polynomial of score against age (years) in male dolphins with error intervals per age category.

Third order polynomial regression resulted in R^2^ values for female and males of 0.97 (Fig 5) and 0.96 (Fig 6) respectively. The following equations can therefore be used to estimate age, with y = age estimation and x = ossification score.

Females: *y* = 0.0000383*x*^3^ − 0.0019398*x*^2^ + 0.1101071*x* − 0.2517343

Males: *y* = 0.0000293*x*^3^ − 0.0010975*x*^2^ + 0.1462751*x* − 1.0465702

The results for the optimal model fit are presented in Table 1. The corresponding test results are presented in Table 2, showing a global RMSE for females and males of 2.4 years. The smallest error was in females less than 5 years of age where the age could be estimated to within 0.35 years. The largest error was in females > 20 years old of 4.3 years.

**Table 1:** Model fit for age estimation in female and male dolphins.

	< 5 years	5–10 years	10–20 years	> 20 years	Global
**Female** R2	0.977	0.972	0.977	0.980	0.969
Adjusted R2	0.975	0.970	0.975	0.979	0.966
RMSE	2.013	2.115	1.870	1.908	2.180
**Male** R2	0.985	0.988	0.987	0.990	0.978
Adjusted R2	0.984	0.988	0.986	0.989	0.976
RMSE	1.838	1.671	1.567	1.588	1.851

**Table 2:** Test phase results showing the RMSE for each age demographic and global RMSE in years for female and male dolphin age estimates.

Age Group	Female RMSE (years)	Male RMSE (years)
<5 years	0.35	0.87
5–10 years	0.88	1.32
10–20 years	1.88	1.82
> 20	4.28	4.09
Global	2.43	2.44

**Fig 7:**
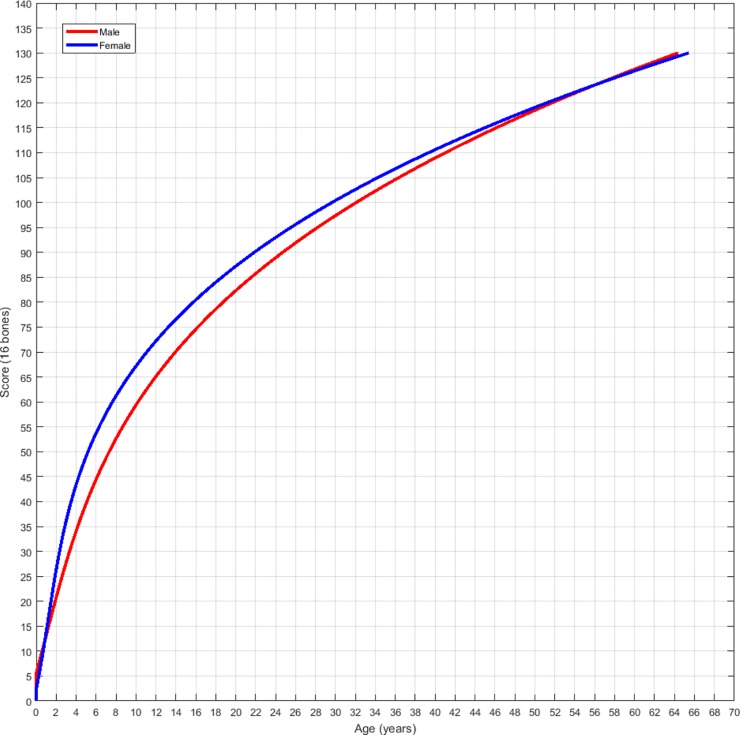
Comparison of the growth rates with female dolphins (blue) growing faster and reaching sexual maturity earlier than male dolphins (red).

In comparison of the scores for a given age between the two sexes the Wilcoxon Signed Rank Test confirmed a significant difference between sexes, with p = 0.00001. Fig 7 demonstrates the comparison of the growth rates with female dolphins (blue) growing faster and reaching sexual maturity earlier than male dolphins (red).

At the studied region, the first epiphyseal center to appear is associated with the distal radius and ulna and appears at approximately 4–6 weeks of age. Table 3 lists the defining changes and the ages at which they occur in males and females. The predictive value of the ossification centers varies with age and changes during growth. The location of the primary focus of assessment should shift according to estimated chronological age. For example metacarpal IV and V are of greater importance in older age. The transition of the radius and ulna from stages 4 to 5 occurs in conjunction with sexual maturity. The distal phalangeal surfaces do not reach stage 5 until several years after the radius and ulna, and metacarpal 5 does not reach stage 6 until even later. The additional lateral carpal bone, adjacent to the ulna and proximal to metacarpal 5 does not develop in any female younger than 30 years and was present only in one male older than 50 years. Consequently, the value of each individual bone carries different weight depending on the age of the individual. The algorithm created can be applied to all ages and therefore includes 16 different measurements. Focusing on degenerative (osteoarthritic or osteolytic) changes will be of greater benefit to accurately estimate age in elderly individuals.

**Table 3:** The defining changes observed radiographically and the ages when these changes are documented in each sex.

Radiographic Observation	Age observed in females	Age observed in males
Secondary Ossification center of radius and ulna reaches full width of opposing diaphysis (Stage 3)	6 months	2 years
Secondary ossification centers present proximally and distally on M IV (Stage 3)	3 years	5 years
Closure of radius and ulna secondary ossification centers (Stage 5)	6–8 years	10–12 years
Metacarpal II proximal closure (Stage 5)	6 years	9 years
Metacarpal II distal closure (Stage 5)	10 years	13 years
2nd Phalanx of M II proximal and distal closure (Stage 5)	10 years	13 years
3rd Phalanx of M II proximal and distal closure (Stage 5)	10 years	14 years
Dissolution of physeal line in radius and ulna (Stage 6)	> 12 years	> 15 years
Dissolution of MII and MIII physeal lines (Stage 6)	> 20 years	> 25 years
Appearance of lateral ulna ossification	> 30 years	> 40 years

## Discussion

Skeletal ossification follows a predictable systematic progression, enabling accurate estimation of chronological age. Although well established in human medicine, previous application to wildlife conservation is limited due to lack of known aged individuals. Previous studies have documented age associated changes in cetacean flippers but have been unable to create a predictive model to estimate age without a control population [38]. Examining the chronological progression of skeletal ossification in this known age population has enabled accurate prediction curves to be created to facilitate age estimation of dolphins of unknown life stage.

Healthy cetaceans of known chronological age are primarily limited to those in human care whose original birth date and life history is well documented. Sexual dimorphism and different growth rates have required two separate formulas to be created, one for each sex [45]. Female dolphins have been shown to grow at a faster rate initially, until they reach sexual maturity, then asymptote in growth earlier than males (Fig 7) [46]. Females are documented to reach sexual maturity at approximately 6–8 years old with a range of 5–12 years of age previously observed [45–47]. Males however take longer to reach sexual maturity with ages of 10–15 years being observed [45]. This is consistent with the ages observed of growth plate closure in this study with ages 6–8 years showing the transition of radial scores from 4 to 5 in females and age 10–12 years showing the same changes in males (Table 3).

In humans, ethnic origin has less effect on skeletal maturation rates than the populations socio-economic status [48]. In cetaceans, species variation in the age of sexual maturity has been linked to both behavior and life span. It may be in dolphins that species variation exists, however this could also be linked to food availability and natural stressors in the geolocation of the population. An example of species variation is in the harbor porpoise, where sexual dimorphism and early sexual maturity results in parallel growth between males and females up until 6 years old, when females show more progressive development [26, 49]. This is thought to be linked to earlier male pelvic bone and musculature development to allowing faster swimming speeds in order to successfully mate with the female [36]. Increases in estrogens and testosterone levels during puberty promote mineralization of growth plate cartilage driving bone maturation [50]. This fact facilitates application of the knowledge of species life span and sexual maturity permitting radiographic interpretation of skeletal ossification to allow extrapolation of this model to other species and provide an approximation of age demographic [51]. Female fin whales (*Balaenoptera physalus*) have been shown to attain sexual maturity later than males but in the vast majority of cetaceans males mature later than females [52].

**Fig 8:**
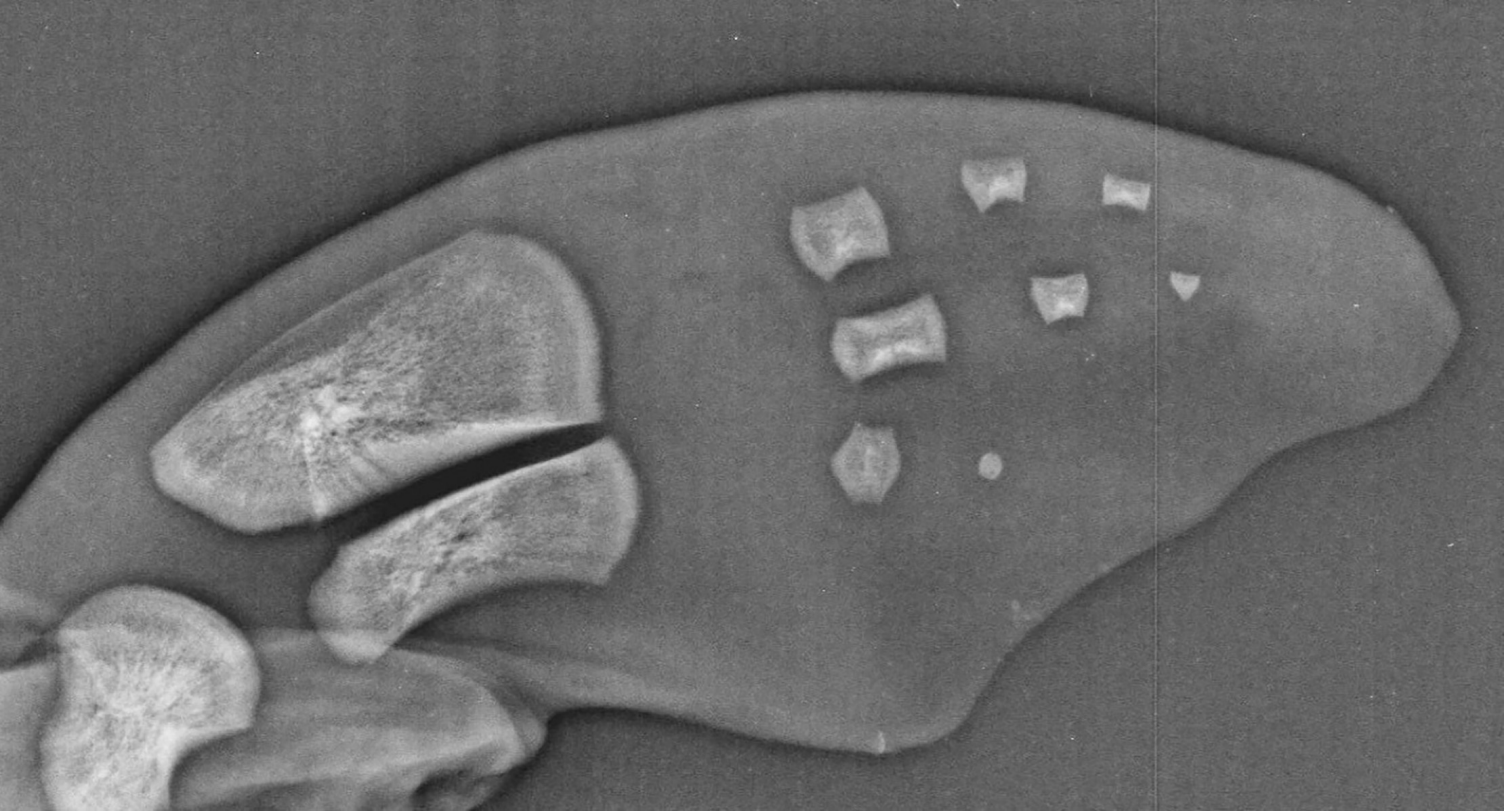
Radiograph of an aborted female fetus at 6 months prior to parturition. Note the lack of secondary ossification centers and missing carpal bones and metacarpals I and V.

Human age estimation curves from hand radiographs cease post age 18, when sexual maturity has completed and epiphyseal lines are obliterated [22]. Due to paedomorphosis and hyperphalangy of cetaceans, progressive age-related changes continue to occur after sexual maturity, enabling application of this skeletal maturation concept to age dolphins of all life stages. The additional inclusion of degenerative changes in the scoring system allows increase accuracy in geriatric animals also, long after skeletal maturation of most bones has completed [25]. The use of negative scoring in the formula facilitates the application of this technique to aid diagnosis of the timing of abortion and determining fetal vs neonatal tissues. In aborted fetuses there will be no primary ossification centers of the carpal bones or distal phalanges and a complete lack of secondary ossification centers (Fig 8). This could facilitate age estimation in decomposing neonatal carcasses where length data is not available [29].

**Fig 9:**
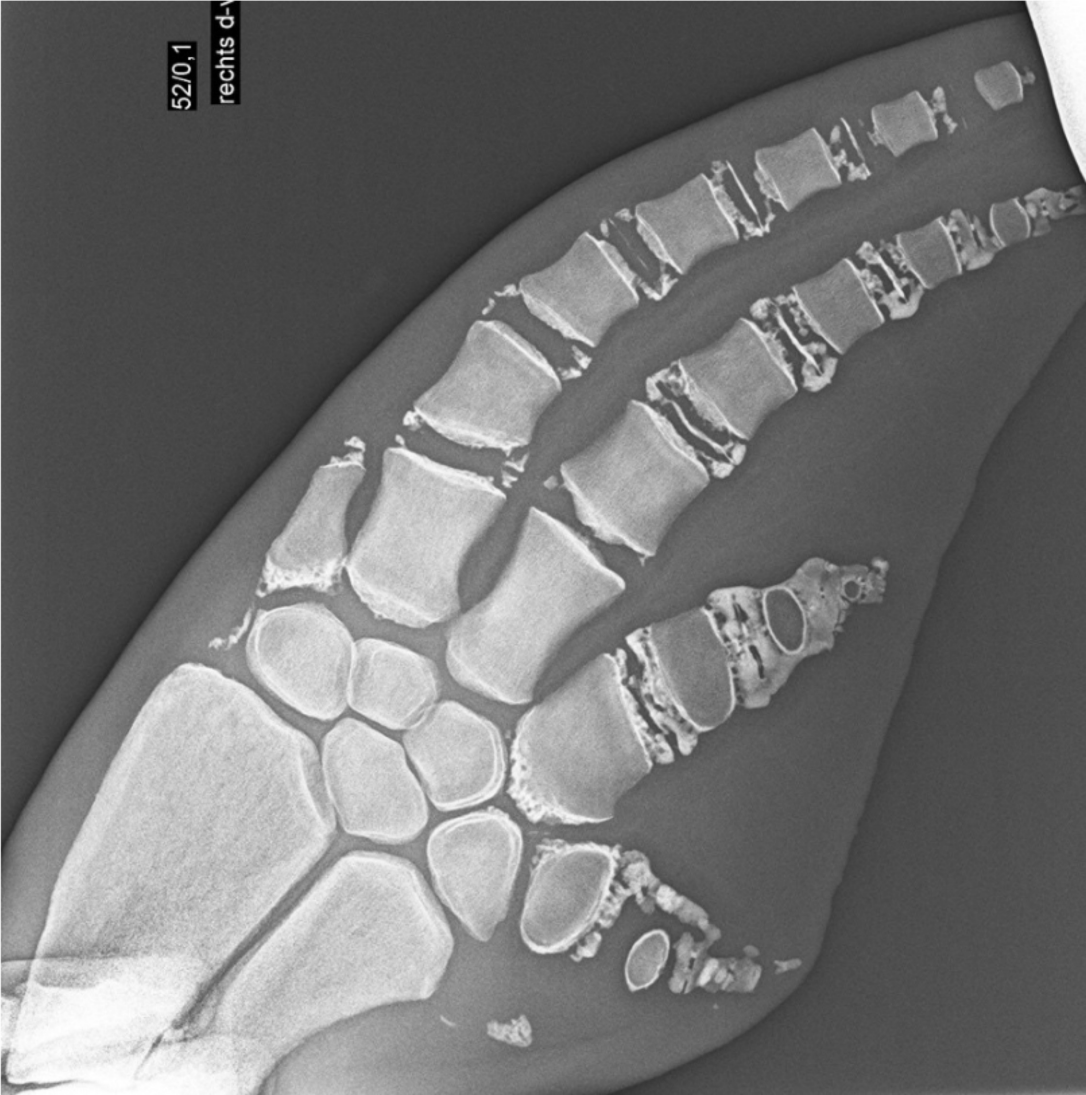
Radiograph of a pectoral flipper of a 58 year old male dolphin. Note the osteoarthritic changes and calcification of the cartilages demonstrating the degenerative changes observed in geriatric dolphins.

Inevitably, acquisition of radiographs of geriatric dolphins of known age was limited. Accurately aging older radiographs relies on documenting osteoarthritic changes including cartilage calcification; Fig 9. Due to the subtle changes occurring in older animals there is increased standard deviation in age estimation > 20 years old compared to animals < 5 years of age, where skeletal ossification has improved precision of predicting the age to within a few months. Skeletal aging is still apparent radiographically in geriatric dolphins, shown by one male included in the dataset where radiographs were taken at age 40 and 49 and progressive aging of the distal phalanges and degenerative changes were easily differentiated. Caution should be taken in suspected older cases as it is possible that the edges of the metacarpals and phalanges become rounded again due to osteolytic degenerative changes so the bone may appear like a score 5 but actually indicate 8. This demonstrates the benefit of interpreting each individual bone within context of the entire radiograph. The life span of free-ranging dolphins is estimated to be around 35 years old in females and approximately 30 years old in males with maximum ages of 60 previously reported [45, 53].

Each individual bone can be assessed discretely, however, one should also aim to interpret the bone score within the context of the radiograph. For example if the growth plate appears to be partially closed, but the alignment of the carpal bones is ambiguous then comparing the proximal surface of the 2^nd^ metacarpal with the distal score of the radius would enable improved interpretation. In dolphins, ossification proceeds along the proximo-distal axis of the metacarpal and phalangeal bones. In addition, fusion of the growth plates of the radius and ulna is known to occur from axial to abaxial and in synchrony, therefore the scores of the radius and ulna are likely to be comparable. Incongruous long bone growth can be indicative of nutritional or metabolic abnormalities [54]. Dolphin carpal bones showed inconsistent signs of ageing and were therefore not included in the final 16 scoring locations. The delta-shaped bones, however, aged in a different series and required their own scoring system to objectively score them (Fig 3) [38]. In humans, delta-shaped metacarpals are referred to as a longitudinal epiphyseal bracket, being a pathological finding [55]. Delayed perichondral ossification is a normal finding in cetaceans and facilitates the use of radiographic age estimation through all life stages.

An advantage of the “atlas” approach to radiographic assessment rather than a scoring system bone by bone for age estimation is the ability to assess the overall picture and observe each bone within context of the next. There is potentially a greater margin for error due to the less quantitative and more subjective methodology. While the individual bone scoring is potentially more laborious, once the reader is familiar with the system, it is very quick and easy to use and can allow for a fixed quantitative estimate to be applied. In considering the advantages and disadvantages of each methodology, this manuscript aims to provide both options to enable age interpretation of a radiograph of a dolphin pectoral flipper[56].

A single radiograph was obtained from each dolphin rather than bilateral pectoral radiographs. This was due to the lack of differences between both flippers in all animals sampled at the beginning of the study and the absence of laterality behavior and asymmetry observed in previous studies between pectoral flippers [4, 25, 37, 40]. Advantages of a single radiograph include a reduction in radiation exposure to the dolphin and the operators and a reduction in time and effort required to obtain a single image rather than two. The practical approach enables the technique to be applied to both live cetaceans, stranded, in the field or in human care, in addition to archived museum specimens. The flat, thin region of the pectoral flipper enables reproducibility and reduced positional variation. Additionally, in the flipper the bone tissue contrasts highly with surrounding soft tissue, allowing low power battery operated radiographic equipment with short exposure times still obtaining adequate sharp diagnostic images. This non-invasive approach is highly advantageous in comparison to the complex tooth GLG methodology, utilizing simple equipment available within a standard veterinary practice [57].

**Fig 10:**
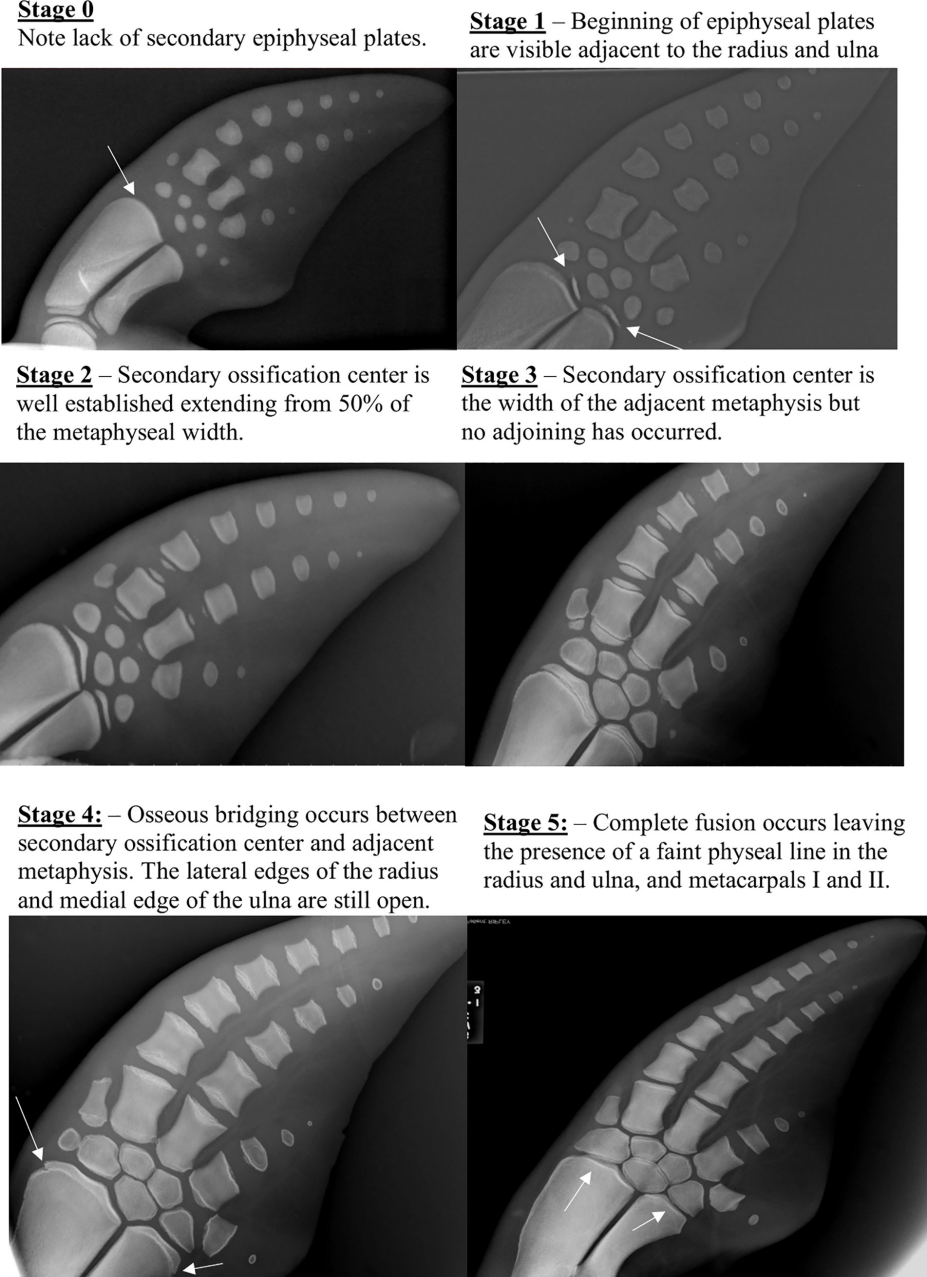
Progressive ageing of the radius and ulna with associated stages.

The factors determining normal maturation patterns are still not fully understood. However, genetics, environmental factors, endocrinological status including thyroxine, growth hormone and sex steroids play important roles [25, 32]. The presence, absence or fusion of certain bones appears to be genetically determined and unrelated to the age of the animal (Fig 10, Stage 7). This could also provide relative information regarding paternities. Discrepancy between skeletal age and dental age has been shown to be linked to possible hormonal abnormalities [58, 59]. Pathology is more likely to slow skeletal maturation and retard growth, however some conditions do exist which can accelerate skeletal development, such as precocious puberty and hyperthyroidism. In dolphins with known endocrinological diseases, such as those with adreno-cortical insufficiency, there could be an impact on the rate of skeletal maturation, potentially resulting in age estimations lower than the actual chronological age [60, 61].

Nutritional status is known to affect bone density with suboptimal nutrition linked to the delayed growth plate closure [62]. In addition to the presence or absence of metabolic disease, alterations of nutritional standards may also influence the growth rate and subsequent radiographic appearance [63]. For example, the negative effect of rickets on growth plate closure in humans is well described [54]. Interpretation of blood analytes such as ALKP in conjunction with radiographic assessment could enable diagnosis of metabolic disturbances which could impact on growth rate and subsequent age estimation [64].

The timing and duration of the nutritional insult is paramount to the long term effects observed, with rapid response to nutritional compromise being observed to reduce the chronic effects [65, 66]. Negative effects of compromised nutrition are likely to be more marked in subadults with open growth plates than in a skeletally mature adult. Stress or trauma have also been represented in the diaphysis of long bones with alterations in growth rate resulting in a stress line of Harris of increased density due to arrested growth [67]. Observations of Harris lines can aid in overall health assessment and provide an estimation of the timing of previously traumatic, stressful or nutritionally compromised events [68]. The transverse scar demonstrates the area of bone which was contiguous with the epiphyseal cartilage at the time of the insult, providing an estimation of the timing of compromised growth. This could potentially be applied to free-ranging dolphins which have had a known traumatic event such as the Deepwater Horizon Oil Spill in 2010 [69]. However the technique can be dependable of good radiographic resolution in order to properly interpret these changes as well as certain details such the extension of ghost physeal line to discern between stages 5 and 6 or to detect initial pathological mineralization’s as degenerative changes.

The radiographs were analyzed by two experienced marine mammal veterinarians, however neither individual had experience specifically in radiography or skeletal maturity determinations. Consequently, interdisciplinary collaboration with a human physician and a biomedical engineer with extensive experience in diagnostic imaging and skeletal maturity determinations ensured a correct analytic approach was performed in application of the human approach to dolphins (Fig 10).

Reviews of radiographs were performed blind with no knowledge of the health status of the individual by one reviewer. Two additional cases not included in the study had abnormally low growth rates and were easily identifiable based on their radiographs, during blind reviews, with zero prior knowledge to the reviewer of potential health concerns. Both individuals (one male of 10 years old and one male of 20 years old) from different institutions demonstrated retarded growth falling consistently behind the curve. These two animals having clinical history of chronic disease, were excluded from the study due to failure to meet the inclusion criteria of no health concerns. These cases are comparable with human literature with chronic health concerns impacting bone development [70]. This indicates the potential of this methodology also as a clinical diagnostic tool in the identification of subclinical nutritional or metabolic compromised clinical states. Diagnosis could also be made via a time series of radiographs identifying suboptimal progression of expected skeletal ossification.

**Fig 11:**
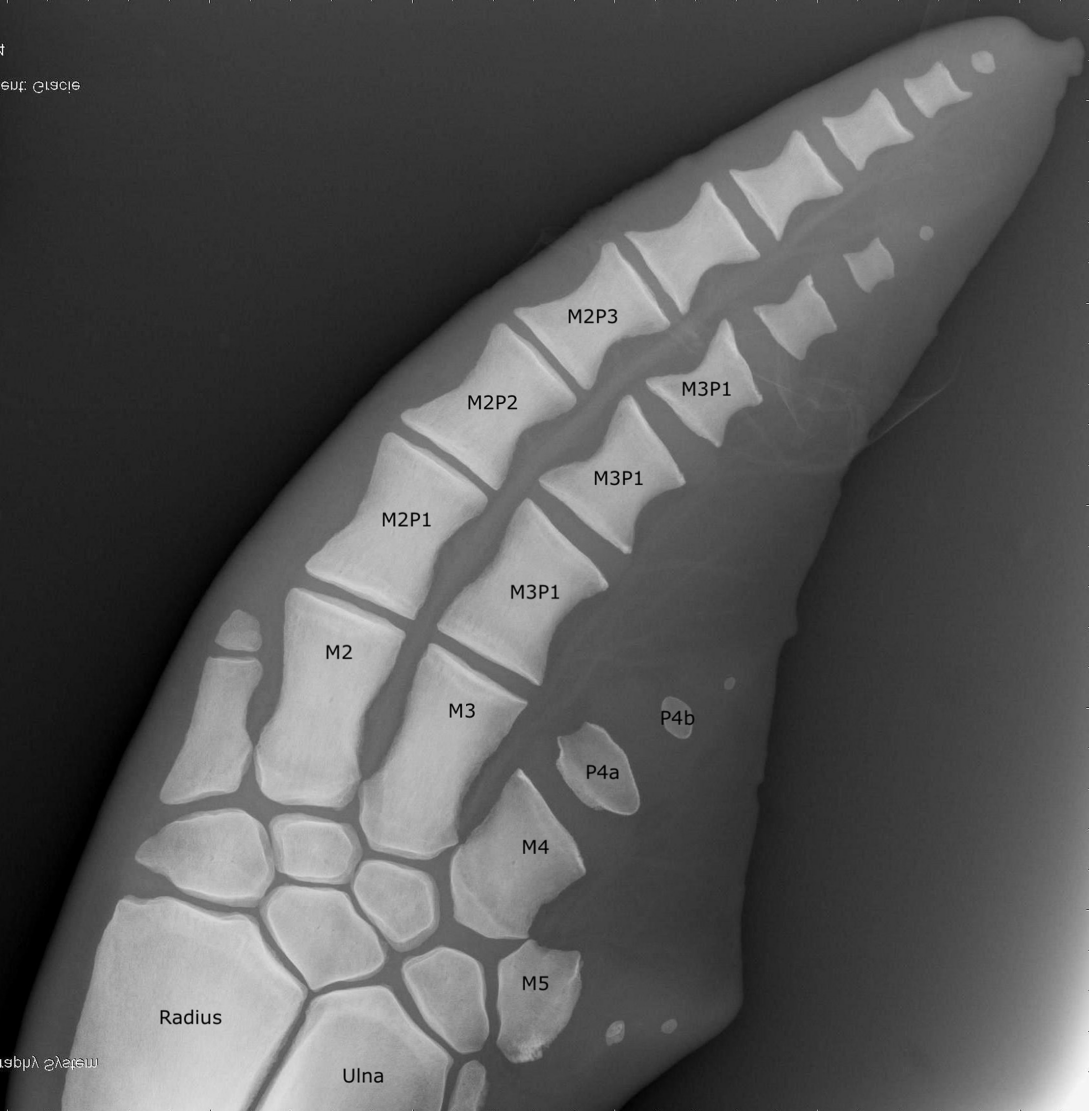
Labelled radiograph to confirm bone identification to enable wide application of the technique if cetacean anatomy is unfamiliar.

The most accurate interpretation of this study of age, growth and development will be highest for those familiar with the technique, cetacean anatomy and sufficient background knowledge in radiography to interpret the images to the full potential of utilizing the tool for health assessment (Fig 11). The use of this method in conjunction with other currently used methods such as body length, dental radiographs, tooth GLG or future methods such as DNA methylation will improve accuracy of the age prediction and facilitate validation. This method not only provides a single age estimation but, potentially more importantly, also provides information regarding the maturation process over time, which is currently unachievable accurately via other methods while still very relevant for understanding natural history in ecological studies. As in human references the arbitrary appearance of the scoring system will be reduced with the more experienced viewers which can interpret the individual bone within the context of the entire flipper [22].

A larger sample size would provide improved accuracy of age prediction. Additional cases to facilitate future model refinement would be welcomed. In any chronological age group of any species there are inevitably individuals with faster or slower rates of physical development for a given chronological age. Skeletal status diversity of individuals of the same age are represented within this cohort enabling the creation of standards which have an accurate standard deviation for a given age. This will enable future application to other populations of bottlenose dolphins. One standard deviation either side of the skeletal age estimation will include two thirds of the dolphin population with two standard deviations including 90%. Outside of this range will likely indicate retarded or accelerated pathological growth abnormalities. Differences in growth rates between free ranging dolphins and those in human care cannot be interpreted at this age. Future research assessing radiographs of a wild population of known chronological age are needed to assess the accuracy of applying these estimation formulas to other populations.

The reproducibility of the radiographic scores (precision) and the accuracy (correct age estimate) both require quality control to ensure a successfully implemented age estimation program. Although in human medicine the single bone scoring method of age determination is more time consuming, it has not been shown to necessarily yield more accurate results than the radiographic atlas, which is why both methods are provided here [56, 71]. Quality control to minimize bias was performed via using known age samples rather than relying on another method to estimate age. The intra and inter observer errors in this study were not significant, indicating precision and reproducibility between reviewers and on multiple assessments of the same image.

Performing 1000 iterations of the model to validate the equation predictions means other dolphins with a comparable genetic and environmental background should correspond closely to the standard created. The inevitable variability amongst individuals of growth rate and final size achieved means some individuals will fit the model better than others. Even in a genetically homogenous population variations will likely occur.

Future research will involve the use of computed algorithms capable of automatically rendering the age of the dolphin from the pectoral flipper radiograph through image recognition with artificial intelligence algorithms [72]. Relatively small sample size and high individual variation in shape and size, coupled with the large number ossification centers for assessment have limited the computer algorithm design. Using the atlas database serves as an intuitive global interpretation via gross visual assessment and direct comparison of cases. Magnetic resonance imaging (MRI) has been shown to provide increased accuracy of age estimation of growth layer closure in older specimens in comparison to radiography. However, for the purposes of field conservation, research and feasibility for the dolphin clinician, radiography is the superior practical option [73].

Previously, despite the availability of radiography in clinical practice and accessibility for ante and post mortem sampling, radiograph interpretation could only ascribe a specimen to a general age class rather than provide an accurate age estimation within 2–5 years [4]. This new technique aims to replace the invasive tooth extraction aging methodology providing a fast readily available alternative. Diagnostically working with images instead of tissues allows simple handling procedures, international sharing of information and widespread use of the technique to improve knowledge of cetacean ages globally. Future progressive inclusion of additional images obtained from animals of known chronological age in light of this database will increase precision and repeatability of age estimations across the demographic. In conclusion, this project not only provides equations to accurately quantify age estimation but also provides a reference collection of images for digital image exchange and to enable collaboration and training among different institutions to extrapolate the principles to other cetacean species.

## Supporting information

S1 Table Individual scores for each radiograph used to create the equations to estimate chronological age. Numbers 1–16 correlate with the 16 anatomical locations outlined in Fig 4. Sum is the total score assigned to each radiograph.
(XLSX)
